# Methods for recombinant expression and functional characterization of human cannabinoid receptor CB_2_

**DOI:** 10.5936/csbj.201303011

**Published:** 2013-09-06

**Authors:** Alexei A. Yeliseev

**Affiliations:** a National Institute on Alcohol Abuse and Alcoholism, National Institutes of Health, 5625 Fishers Lane, Bethesda, MD 20892, USA

**Keywords:** Cannabinoid receptor CB_2_, recombinant expression, purification, functional reconstitution, G protein activation

## Abstract

Cannabinoid receptor CB_2_ is a seven transmembrane-domain integral membrane protein that belongs to a large superfamily of G protein-coupled receptors (GPCR). CB_2_ is a part of the endocannabinoid system that plays vital role in regulation of immune response, inflammation, pain sensitivity, obesity and other physiological responses. Information about the structure and mechanisms of functioning of this receptor in cell membranes is essential for the rational development of specific pharmaceuticals. Here we review the methodology for recombinant expression, purification, stabilization and biochemical characterization of CB_2_ suitable for preparation of multi-milligram quantities of functionally active receptor. The biotechnological protocols include expression of the recombinant CB_2_ in *E. coli* cells as a fusion with the maltose binding protein, stabilization with a high affinity ligand and a derivative of cholesterol in detergent micelles, efficient purification by tandem affinity chromatography, and reconstitution of the receptor into lipid bilayers. The purified recombinant CB_2_ receptor is amenable to functional and structural studies including nuclear magnetic resonance spectroscopy and a wide range of biochemical and biophysical techniques.

## Introduction

Heptahelical G protein-coupled receptors (GPCR) are integral membrane proteins involved in a wide array of cell signaling pathways. The cannabinoid receptor CB_2_ that belongs to the rhodopsin-like (class A) GPCR is an attractive target for the development of drugs for management of pain, inflammation and immunological disorders [1–3]. Structural studies will provide critical insights into the molecular mechanisms of ligand binding and signal transduction, and can contribute to the rational design of novel specific drugs targeting this receptor.

The progress in structural studies of GPCR has been relatively slow until recently, primarily due to (i) difficulties in obtaining large quantities of sufficiently pure, homogenous and functional receptors, (ii) conformational flexibility of GPCR that hinder their stabilization in detergent micelles, and (iii) high hydrophobicity of these integral membrane proteins that complicates preparation of well-diffracting crystals for X-ray crystallography. In spite of these obstacles, significant improvements in expression techniques, methods of stabilization and crystal preparation resulted in several high resolution structures of GPCR solved during the past few years [4–12].

With the notable exception of rhodopsin, most GPCR are present in native tissues at relatively low levels, and recombinant expression in a heterologous host is currently the only practical way to obtain these proteins in milligram quantities necessary for structural studies. The commonly used expression systems for GPCR include baculovirus-infected insect cells, yeast, bacterial or mammalian cells as well as cell-free systems [6]. Expression in insect cells has been particularly useful for production of receptors in milligram quantities for crystallization trials [5, 9, 10, 13, 14]. However, the adaptation of insect- or mammalian cells for preparation of stable-isotope labeled proteins for nuclear magnetic resonance (NMR) spectroscopy studies currently is prohibitively expensive due to the complexity of the medium and high cost of labeled nutrients [15]. While the expression in yeast cells has also been used for production of several GPCR [16, 17], this host may not be suitable for some receptors (including CB_2_) because it is often prone to non-homogenous glycosylation or partial proteolysis of target proteins [18–20].

These considerations stimulated the development of methods of production of the recombinant CB_2_ in *E. coli* cells. In a series of publications we reported efficient expression of CB_2_ receptor in bacterial cell membranes in a fully functional form, although in the absence of posttranslational modifications [21], and its purification to over 90% homogeneity by tandem affinity chromatography [22]. Furthermore, the protein can be labeled with stable isotopes by fermentation of *E. coli* in a defined-composition medium supplemented with labeled nutrients [23]. This robust methodology for expression and labeling of CB_2_ opens up exciting opportunities to study this receptor by NMR spectroscopy.

In addition to the availability of milligram quantities of purified receptor, structural methods require sufficient stability of the protein over extended periods of time. While solubilization in detergents is needed for isolation of GPCR from cell membranes, preventing irreversible denaturation of these proteins in detergent micelles is a notoriously difficult task [6–8, 13, 24–26]. Here we review the methodological approaches for stabilization and reconstitution of the purified receptor in lipid bilayers and preparation of milligram quantities of functional CB_2_ suitable for studies by a broad array of biophysical techniques.

## Experimental design

Several laboratories reported expression of either full-length or truncated human cannabinoid receptors in a heterologous host including bacteria, yeast, baculovirus-infected insect cells and cell-free system [18–20, 27–32]. While production of a ligand binding-competent receptor was demonstrated, no successful attempt was reported to produce and purify the expressed receptor in large quantities, to label it with stable isotopes and to stabilize it in a functional form suitable for biophysical studies.

**Figure 1:**
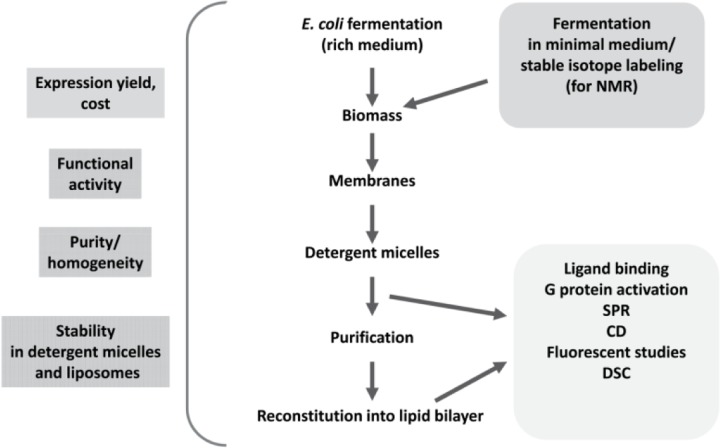
Experimental strategy for preparation of functional CB_2_ (adapted from [24]).

A comprehensive program initiated in our laboratory has an objective to develop an extensive set of methods for recombinant expression of human cannabinoid receptor CB_2_ in large quantities in *E. coli*, efficient purification, stabilization in detergent micelles, and functional characterization. Various elements of this methodology were reported in several earlier publications [21–24, 33, 34], and the general outline of the experimental strategy is given in Figure 1. The initial stage of the study focused on establishing conditions for production of the functional CB_2_ receptor in *E. coli* cells cultivated in a rich 2xYT medium while subsequent work dealt with the adaptation of expression protocols to preparation of stable-isotope labeled receptor by fermentation in minimal salt media of defined composition. Particular attention was devoted to maximizing the recombinant protein yield, reducing the cost of fermentation, stabilization of the functional CB_2_ receptor in detergent micelles and achieving high purity and homogeneity of protein preparations. In parallel, methods for functional analysis of the purified receptor by ligand binding and G protein activation (either in detergent micelles or reconstituted in lipid bilayers) were developed. Furthermore, the purified CB_2_ was characterized by several biophysical techniques including NMR spectroscopy, surface plasmon resonance, CD-, IR- and fluorescent spectroscopy, and differential scanning calorimetry.

## Expression of CB_2_ receptor in *E. coli*

The choice of the bacterial host, copy number of the expression vector, strength of the promoter, composition of the culture media, concentration of the inducer as well as temperature, method and duration of induction play critical roles in determining both the total yield of the fusion protein and the recovery of functional receptor. Several *E. coli* strains including BL21 (DE3), DH5a, KRX, RosettaGami, C41 and C43 were compared for their effectiveness in production of fusion CB_2_, and BL21 (DE3) was selected based on higher yield of the recombinant protein and high levels of functional activity of the receptor [21, 22, 35].

Limited availability of tRNAs for rarely used codons may play a role in controlling the rate of translation of the recombinant polypeptide [36, 37]. We optimized the codon usage of the human CB_2_ gene for bacterial expression by designing a synthetic gene enriched with synonymous codons reported to be frequently used in *E. coli* [38]. However, the use of the synthetic gene did not increase the yield of CB_2_ when introduced into the expression vector under the control of the *lac* promoter; it even resulted in slightly lower levels of the recombinant receptor compared to the original mammalian gene sequence (Yeliseev et al, unpublished). This suggests that the rate of folding and insertion of CB_2_ in the bacterial membrane rather than the rate of translation has a critical influence over the yield and correct fold of the recombinant receptor. Therefore, in all subsequent experiments the native sequence of the human CB_2_ gene was used.

**Figure 2:**
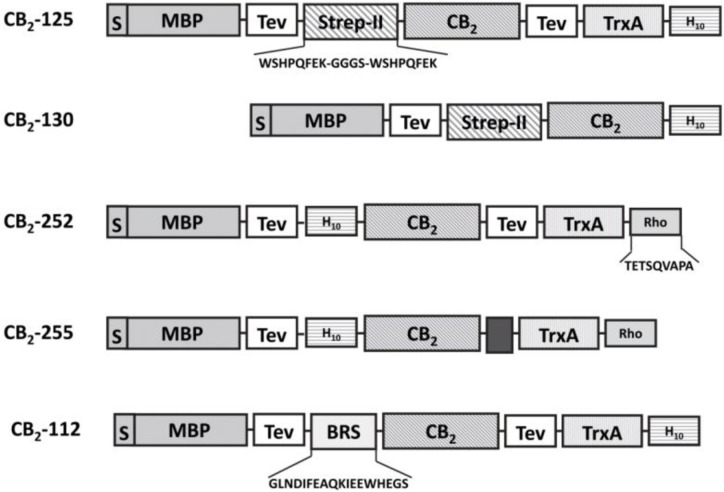
Schematic representation of constructs for expression of CB_2_ fusion protein in *E. coli* (adapted from [22, 33, 39]).

The nature and relative position of expression partners fused to the target protein can change dramatically the expression level and activity of the recombinant GPCR [6]. Therefore, a variety of plasmid constructs containing different combinations of several expression- and solubility tags were tested for their efficiency in production of functional CB_2_. Figure 2 depicts selected expression constructs currently utilized in our laboratory.

To avoid misfolding and aggregation of the recombinant CB_2_, and to ensure its expression in a functional form in *E. coli*, an appropriate N-terminal fusion partner is required [22]. For example, the N-terminal Haloalkane dehalogenase (Halotag) significantly increased the levels of the fusion CB_2_ protein but a large fraction of the receptor was not functional (likely due to misfolding and aggregation) [35]. On the other hand, the use of the maltose-binding protein of *E. coli* (MBP) fused at the N-terminus of CB_2_ was highly beneficial for the high-level functional expression of this receptor [21, 22, 28]. The MBP is normally localized in the periplasm of *E. coli*, and the transport of the nascent polypeptide across the cytoplasmic membrane is facilitated by the Sec translocon system that recognizes the leader sequence of MBP [40]. Importantly, the full length sequence of MBP is required for the maximal beneficial effect. Expression of CB_2_ fused to only the 26 amino acid-long leader sequence resulted in a significantly lower (∼50-100-fold) expression and activity suggesting that the whole-length MBP is highly beneficial to ensure the correct fold of CB_2_ in *E. coli* membranes [22]. A recent study reported the use of N-terminal Mistic (from *Bacillus subtilis*) and C-terminal TarCF (fragment of bacterial aspartate chemosensory transducer) for functional expression of CB_2_ in *E. coli* [31]. However, the density of ligand-binding sites in cell membranes was an order of magnitude lower than that of MBP-containing expression constructs [21].

The topology of the receptor in cytoplasmic membranes was determined by measuring the *in vivo* biotinylation of the fusion protein containing a biotin-recognition sequence placed in various positions relative to CB_2_ [33]. When expressed as a fusion with MBP, the N-terminus of CB_2_ is exposed to the periplasmic space while its C-terminal part is localized to the cytoplasm of *E. coli*. The predominant *in vivo* biotinylation of the C-terminal biotin-recognition sequence suggests that MBP promotes the insertion of the fusion protein into cytoplasmic membranes in an N-terminus-out orientation. Furthermore, one can speculate that the oxidative redox environment of the periplasm facilitates the formation of the proposed disulfide bond between the cysteine residues 174 and 179 in extracellular loop 2 [33] which may have an important role in stabilizing the functional fold of CB_2_ [41]. The expression of CB_2_ can be further increased by 30-40% by fusing the *E. coli* thioredoxin (Trx) to the C-terminus of CB_2_ [22]. Both MBP and Trx expression partners can be selectively removed if desired by cleaving the fusion with the specific TEV protease at recognition sequences flanking the receptor.

For purification by tandem affinity chromatography, two small affinity tags were added at the opposing ends of the recombinant CB_2_ receptor [22, 33, 39]. Examples of several such constructs are shown in Figure 2. The affinity tags either can be removed in the course of purification or left fused to the purified receptor, depending on the requirements of downstream applications. Examples of the efficient purification of CB_2_ include sequential Ni-NTA and StrepTactin affinity chromatography (for His- and Strep-tag pair) [22], His-tag/ Bio-tag [33] and Rho-tag/ His-tag pairs [39].

Proper alignment of the rates of protein synthesis with the capacity of cells for correct folding and membrane insertion of the nascent polypeptide is essential for production of the functional receptor [23]. Excessively high rates of protein synthesis typically lead to misfolding of membrane proteins and formation of inclusion bodies. Therefore, the expression of CB_2_ is performed from the low copy number vector based on the pMal-p2 backbone from the weak *lac* promoter as described earlier [21].

**Figure 3:**
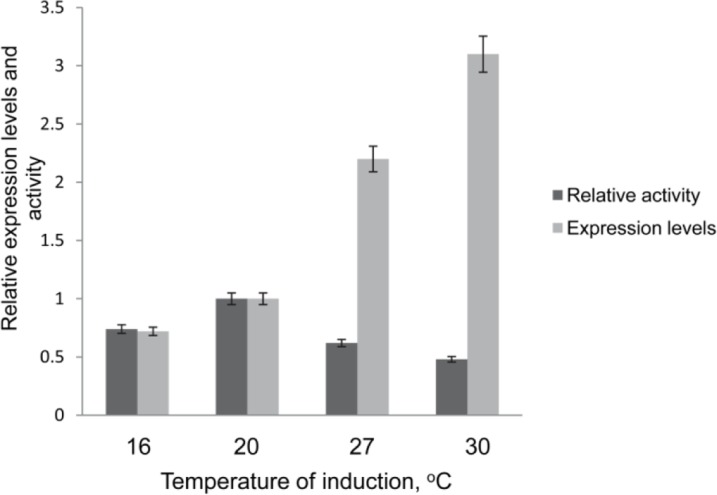
Expression levels and activity of CB_2_ in *E. coli* membranes as a function of incubation temperature. The levels of CB_2_ in membranes were quantified by Western blot, and activity – by a G protein-activation assay. Relative expression levels and activity of the receptor induced at 20 °C were set at 1 (adapted from Ref. [23]).

Likewise, lowering the cultivation temperature from 37 °C to 20 °C has a clear beneficial effect for the functional expression of CB_2_ (Figure 3) [23]. However, further lowering the temperature to 16 °C has negative consequences because the accumulation of the target protein decreases substantially. On the other hand, the cultivation at higher than 20 °C temperature (27°C or 30°C) resulted in a 2-3.5 fold increase in the levels of the fusion CB_2_ in membranes but a large fraction of the accumulated receptor was not functional [23]. Therefore, an induction temperature of 20 °C was deemed optimal for shake-flask experiments.

The typical expression protocol for production of recombinant CB_2_ includes cultivation of *E. coli* BL21 cells harboring an expression plasmid, in shake-flask, in a double-strength YT medium supplemented with 0.5-1% glucose to adequately support cell growth. Cells are grown in 2L baffled flasks containing 500 mL of medium and antibiotic, at 230 rpm to maintain adequate aeration level, and the target protein production is induced by addition of 0.5 mM IPTG, at 20°C. The cultivation then continues for another 40 hours, and the expression levels of CB_2_ can reach 0.5 mg/ L of culture or higher, depending on a construct used [21, 22].

For stable-isotope labeling of CB_2_ the expression in a minimal salt medium (MSM) in a fermentor was developed. *E. coli* cells harboring the expression construct have to undergo at least three rounds of adaptation to the MSM that results in the cell doubling time of less than 2 hours. The cultivation is performed under controlled pH, temperature and aeration [23]. Either glucose and ammonium salts or a mixture of amino acids can be used as sources of carbon and nitrogen. The rates of accumulation of the recombinant receptor in cells cultivated in a fermentor are usually higher than in shake flasks, and the biomass at the OD_600_ = 20-30 or higher can be collected as early as 4-10 hours post-induction, depending on conditions of fermentation and the expression construct.

## Purification of recombinant CB_2_

Purification of CB_2_ and removal of expression partners is typically performed following solubilization of the fusion protein in detergent micelles. By screening solubilization conditions with ∼ 40 different detergents and combination of detergents, a mixture of the nonionic dodecyl maltoside (DDM) and zwitterionic CHAPS was identified as the most suitable for efficient extraction of the CB_2_ from membranes [21, 24]. This detergent mixture is further supplemented with a derivative of cholesterol, cholesteryl hemisuccinate (CHS) and a high affinity ligand (agonist CP-55,940 or inverse agonist SR-144,528) to stabilize the receptor [24]. Use of DDM, CHAPS and CHS for solubilization and purification was also described for neurotensin receptors and other GPCR [26, 42]. A possible mechanism of stabilization by CHS of the recombinant adenosine A_2A_ receptor in DDM micelles has been proposed [43].

The relatively high concentration of the non-ionic detergent DDM (1% w/v) and zwitterionic CHAPS (0.5% w/v) is needed for efficient solubilization of CB_2_ from bacterial membranes [24]. However, the subsequent chromatographic purification can be performed at a lower concentration of DDM (0.1%), which in combination with 0.5% CHAPS and 0.1% CHS [21], is sufficient to maintain the CB_2_ receptor in a correctly folded, soluble form [21]. Since the fusion CB_2_ is accumulated in *E. coli* membranes at moderate levels (∼0.1% of total cellular protein), a very efficient and selective chromatographic procedure is required to achieve the yield and purity of the target protein required for high resolution structural methods. Such commonly used chromatographic techniques as ion-exchange, size-exclusion or hydrophobic chromatography were not efficient, likely because large detergent micelles prevented proper interaction of the receptor with the resin (Yeliseev et al, unpublished). Efficient purification and recovery of CB_2_ was achieved by tandem affinity chromatography taking advantage of small affinity tags placed at opposing ends of the receptor (Figure 2). This approach relies on a tight binding of the affinity tags to their respective resins, allows fast removal of impurities and products of truncation of CB_2_, and results in highly pure, full-length receptor [35, 39].

A typical purification protocol for fusion constructs CB_2_-125 and CB_2_-130 (both containing C-terminal decahistidine tags) [22] begins with the IMAC chromatography. The binding of a shorter histidine tag (i.e. hexahistidine) to the Ni-NTA resin in the presence of detergent micelles was shown to be rather weak [21, 42]. The longer, decahistidine tag makes the Ni-NTA purification step much more efficient and typically results in a recovery of ∼80% or more of the fusion CB_2_, with ∼70% or higher purity [22]. The protein is eluted from the Ni-NTA resin in a small volume (several milliliters), and the expression partners can be removed from the fusion by enzymatic cleavage with purified recombinant TEV protease [22, 44]. The fusion protein sample needs to be dialyzed prior to cleavage, in order to lower the content of salts, imidazole (used for elution of CB_2_ from the Ni-NTA resin) and glycerol, that inhibit proteolysis [22]. For more efficient cleavage, the NaCl concentration should be lowered to 100 mM or less and glycerol – to 15% or less. The reaction is performed for at least 4 hours or overnight at 4 °C [22]. While the reaction rate is much higher at higher temperatures, an increase in temperature above 4 °C is not recommended because it promotes irreversible denaturation of CB_2_ [24].

The CB_2_ released from the fusion is then re-captured via the remaining affinity tag (the N-terminal StrepTag in the case of CB_2_-125 or CB_2_-130), and separated from TEV protease, cleavage products and other impurities. Similar to the Ni-NTA chromatography step, capture of CB_2_ on a StrepTactin Macroprep resin is performed in the presence of DDM, CHAPS and CHS. Since the affinity of a single Strep-tag II (WSHPQFEK) to the resin in the presence of micelles is rather weak, a double repeat of this tag is attached to the N-terminus of CB_2_ to ensure a more efficient capture [22]. This double tag typically improves the recovery of the receptor to greater than 70-75%, and the purity – to greater than 90% [22]. An overall yield of the purified receptor can be as high as 0.3-0.5 mg/ L of the shake flask culture, depending on a particular construct and culture conditions.

The purification of CB_2_ using either His-tag/ Bio-tag or His-tag/Rho-tag pairs was described [33, 39]. The Bio-tag and Rho-tag have higher affinity than the His-tag to their respective resins which makes capture of the fusion CB_2_ protein from dilute solutions more efficient.

## Stabilization of CB_2_

**Figure 4:**
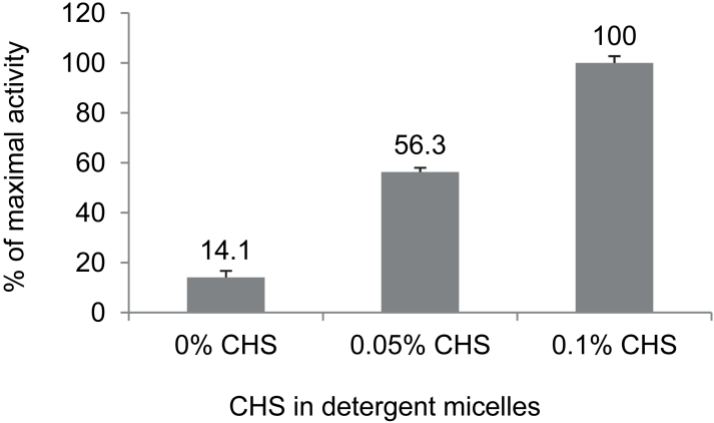
Stabilization of CB_2_ by CHS. Stability of CB_2_ in detergent micelles at 4 °C was tested by measuring functional activity of CB_2_ reconstituted into proteoliposomes from detergent micelles with indicated content of CHS. The activity recovered from micelles supplemented with 0.1% CHS was set at 100%. The receptor was incubated in buffers with indicated content of CHS overnight at 4 °C, and reconstituted into POPC/POPS/CHS lipid matrix in a form of proteoliposomes. The functional activity was determined by measuring the rates of G protein activation by the liposome-reconstituted receptor in the presence of a saturating concentration of agonist CP-55,940. Representative data from a typical experiment (out of a total of 3) are presented; each point is an average of two measurements. (Data reproduced from [24]).

Recombinant CB_2_, like many other GPCR, is highly unstable once solubilized from lipid membranes in detergent micelles. The use of the “mild” nonionic detergent DDM and zwitterionic CHAPS was not only efficient for solubilization but also beneficial for maintaining the functional fold of CB_2_ in micelles for a relatively short period of time [24]. However, structural integrity of the receptor subjected to prolonged exposure (2-3 days) to detergents can be significantly compromised if no additional stabilization is provided [24]. It was established that stabilization with the cholesterol derivative, CHS is essential to protect the correct fold of CB_2_. Depending on a duration of exposure of CB_2_ to detergents, 0.1% (w/v) CHS can significantly increase the recovery of active receptor under these conditions while without CHS the receptor may lose activity rapidly within hours (Figure 4) [24]. Recovery of the functional activity was determined upon reconstitution of the receptor into POPC/POPS lipid bilayers, by measuring the rates of G protein activation by CB_2_ treated with the full agonist CP-55,940 as described [24]. These data correlate well with the reported ∼0.11% CHS required for preservation of the ligand-binding capacity of another class A GPCR, the adenosine A_2a_ receptor [43]. While the mechanism of stabilization by CHS is not yet fully understood, it was speculated that the binding of this lipid-like molecule to the receptor restricts its conformational flexibility and prevents irreversible denaturation [43]. Phospholipids, in particular negatively charged POPS and DOPS, are also very effective in stabilizing the functional fold of CB_2_ in detergents [24].

While CHS is quite effective for stabilization of CB_2_ in micelles, it cannot entirely protect the receptor from irreversible unfolding, especially for prolonged periods of time [24]. Thus, the addition of a high affinity ligand is required for further stabilization of CB_2_. For example, 10 µM CP-55,940 in DDM/CHAPS/CHS micelles increased the yield of the functional protein by up to3-fold (Table 1). Low affinity ligands such as 2-AG were less efficient, while in the absence of ligands a significant fraction of active protein is lost [24].

**Table 1:** Stabilization by high affinity ligands in cell membranes and detergent micelles. (Data adapted from [24]). Ligands at a concentration 10 µM were introduced into buffers during the purification as indicated. Functional activity of purified CB_2_ was determined by comparing the rates of G protein activation of the liposome-reconstituted receptor with that of the fusion CB_2_-130 in *E. coli* membranes used as an activity standard. The measurements were performed at saturating concentration of an agonist CP-55,940 as previously described [24]. To study the effect of ligands in growth media on recovery of the functional CB_2_, 2.5 µM CP-55,940 was added to one culture flask while in the other flask recombinant protein was expressed without ligand. The CB_2_ from both cultures was purified in the presence of 10 µM CP-55,940, reconstituted into liposomes, and the functional activity measured by G protein activation assay as described [24].

Sample	Ligand during protein expression	Ligand in detergent micelles during purification	CHS in detergent micelles during CB_2_ purification,% (w/v)	Specific activity of CB_2_ (%)
**Control (CB_2_ in *E. coli* membranes)**	no	n/a	n/a	100
	no	no	0.1	21±2.3
**Purified CB_2_ reconstituted into liposomes**	no	10 µM CP-55,940	0.1	58±3.7
	no	10 µM SR-144,528	0.1	56±4.3
	no	10 µM 2-AG	0.1	24±3.4
	2.5 µM CP-55,940	10 µM CP-55,940	0.1	98 ±2.5

All cannabinoid ligands including endogenous, plant-derived and synthetic cannabinoids are highly hydrophobic and partition readily into lipid membranes and detergent vesicles. We determined that for the highest recovery of active receptor, the ligand-receptor complex should be formed while CB_2_ still resides in *E. coli* membranes. This can be achieved by adding ligands (CP-55,940 or SR-144,528) during the induction of recombinant protein synthesis (Table 1) [24].

In summary, select non-ionic and zwitterionic detergents, CHS and high affinity ligands have beneficial effects on protecting the functional structure of CB2, and their concerted action is necessary for isolation of fully functional receptor in a purified form.

**Figure 5:**
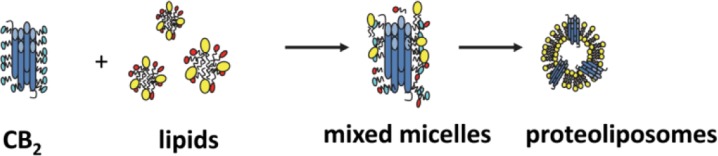
Preparation of proteoliposomes containing purified CB_2_.

To further improve the long-term stability of the CB_2_, the purified receptor is reconstituted into lipid bilayers in a form of small spherical particles (proteoliposomes) [24, 34]. Proteoliposomes can be prepared by removal of detergents using detergent-absorbing resin or by rapid dilution of the lipid-protein-detergent mixture to below the CMC of the detergent (Figure 5). These particles typically have an average size of 120-130 nM, their size distribution is quite narrow, and the protein-to-lipid ratio is uniform within the proteoliposome fraction [34]. Typically, proteoliposomes are quite stable at 4 °C, so that the activity of the receptor in lipid bilayers can be maintained for at least two weeks at 4 °C. Functional activity was quantified by measuring the rates of G protein activation by the agonist-treated CB_2_ and comparing them with that of the receptor in membrane preparations of *E. coli* [24]. The functional receptor in proteoliposomes was preserved for at least several months when stored at -80 °C [24].

The developed methods for expression, stable isotope labeling, purification, stabilization and liposome reconstitution open a path to meaningful NMR spectroscopic studies on the CB_2_ receptor. For example, a solution NMR can be applied to studies of the structure and interaction of CB_2_ with ligands in detergent micelles [21, 45]. However, the experimental setup can be complicated due to relatively short lifespan of the CB_2_ receptor in detergents, especially in micelles devoid of stabilizers (CHS and high affinity ligands). Rather, solubilization in isotropic bicelles [15, 46] or reconstitution into lipid bilayers in the form of proteoliposomes is preferred since a lipid environment better protects the functional structure of CB_2_. Proteoliposomes allow control over the composition of lipid matrix, provide long-term stability for the receptor and generate a native-like environment for studies of allosteric modulation of the functional state of GPCR [47]. For many solid state NMR applications 2-6 mg quantities of the liposome-reconstituted receptor may be required [34].

In addition, reconstitution of receptors into small lipoprotein particles (nanodiscs) is gaining increasing attention since such a system provides easy access to both extra- and intra-cellular surfaces of a GPCR and allows studies of interaction with G protein, β-arrestin and specific antibodies [48–53].

## Functional characterization of CB_2_

**Figure 6:**
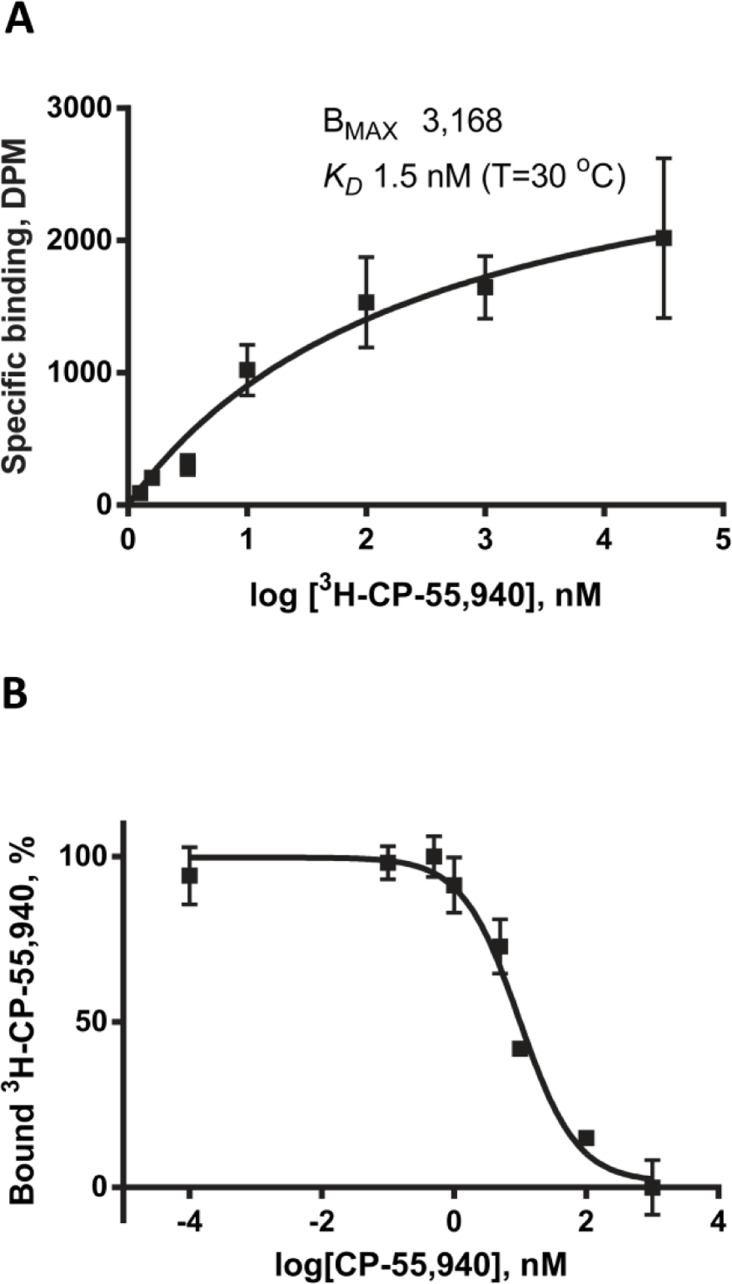
Saturation (A) and competition (B) binding assay with [^3^H]-CP-55,940 on *E. coli* membranes expressing CB_2_-130. Twenty micrograms of membrane preparations containing CB_2_ were used per reaction, and the assay was performed as described in [21]. Each point represents an average of duplicate measurements (adapted from [21]).

Quantitative, reliable tests for the functional activity of the receptor are required for development of techniques of expression, stabilization, and purification of CB_2_. Measurement of binding of specific ligands to the recombinant receptor is typically used to assess the functionality of GPCR in cell membranes. Competitive displacement of the radiolabeled ligand [^3^H]-CP-55,940 with various cannabinoid agonists, antagonists and inverse agonists was performed on a bacterially expressed CB_2_ and revealed remarkable similarities in binding affinity and selectivity to that of CB_2_ in mammalian cell membranes [21]. The methodology for ligand binding on CB_2_-containing membranes was published earlier [21], and typical saturation and competition ligand binding curves are presented in Figure 6.

**Figure 7:**
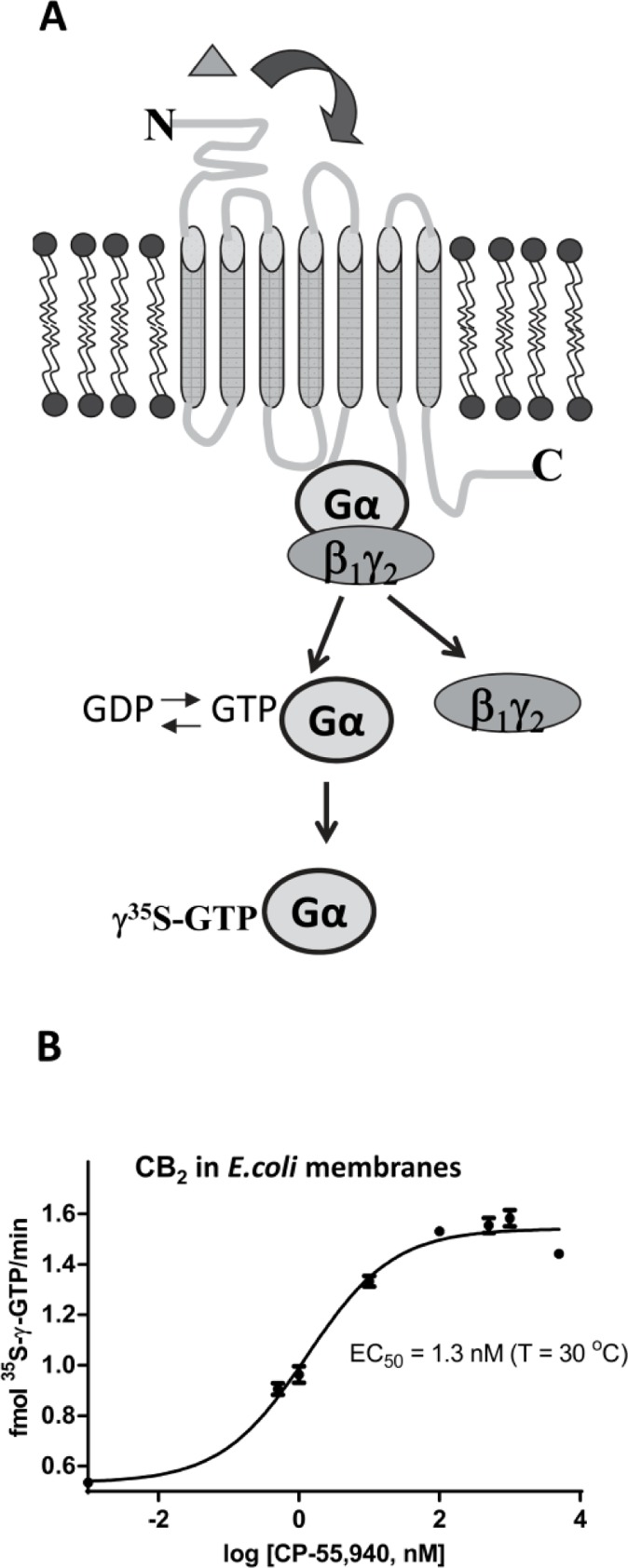
Functional characterization of CB_2_ by G protein-activation assay. (A), a complex of G_α_ subunit of G protein with the non-hydrolyzable analog of GTP is generated upon activation of G protein on an agonist-bound receptor. The rates of accumulation of the ^35^S-γ-GTP-G_αi1_ complex are proportional to the rate of activation of G protein on a receptor; (B), CP-55,940 dose-dependent activation of G protein by CB_2_-130 in *E. coli* membranes. Concentration of components: 3 nM CB_2_, 100 nM G_αi1_, 200 nM G_β1γ2_ in the presence of ^35^S-γ-GTP and variable concentrations of CP-55,940 solubilized in 10 mM MOPS pH 7.5 supplemented with 0.1% BSA in 50 µL reaction volume as described in [21, 24]. Reactions were started by addition of the radiolabeled analog of GTP, proceeded for 20 minutes at 30°C, and were terminated by addition of 2 mL of the ice-cold stop solution (Tris/MgCl_2/_NaCl). Reactions were then filtered through 0.45 µ nitrocellulose filters and retained radioisotope counted upon addition of scintillation liquid (adapted from [24]).

Additional complication comes from the fact that most cannabinoid ligands strongly partition into lipid bilayers or micelles which hampers determination of binding parameters. In particular, nonspecific retention of radioligand on proteoliposomes can reach as much as 80-90% of total binding [24]. To circumvent this problem, another functional test was developed that measures the rates of activation of G protein by an agonist-bound CB_2_ [22, 29]. The activated G_α_ subunit dissociates from the receptor and catalyzes the exchange of GTP for GDP (Figure 7, A). By using radioactively labeled non-hydrolyzable analog of GTP, ^35^S-γ-GTP, the rates of activation of G protein can be readily measured; this, in turn, provides valuable information about the functional state of the receptor [22, 24]. The subunits of G protein can be expressed and purified as described [29]. This test utilizes mostly water-soluble components and is typically characterized by excellent signal-to-noise ratio [24].

The G protein activation test has been routinely used to assess the functionality of CB_2_ expressed as a fusion with MBP and affinity tags in *E. coli* membranes. The presence of N-terminal MBP and the C-terminal Trx and small affinity tags such as His-tag do not affect the rates of activation of G proteins on an agonist-bound receptor [22, 24]. The *E. coli* membranes are devoid of endogenous G protein, and thus contribute very little to the signal measured by the amount of G_α_ -^35^S-γ-GTP. The activation of G protein on *E. coli* membranes expressing CB_2_ upon titration with the high affinity agonist CP-55,940 occurs in a concentration-dependent manner with the estimated EC_50_ = 1.3 nM (T = 30 °C) (Figure 7, B). The EC_50_ for CP-55,940 measured on CB_2_ expressed in CHO cell line was very similar (EC_50_ = 1.37 nM, T= 30 °C) to the values obtained for the bacterially expressed receptor, confirming full functionality of the MBP-CB_2_ fusion in *E. coli* membranes [24].

While the CB_2_ in membrane preparations exhibits robust activation of G protein in response to agonist binding, this assay cannot be performed on a receptor solubilized in detergent micelles since detergents disrupt the interaction of G protein with CB_2_ [24]. Therefore, the purified CB_2_ is reconstituted into small proteoliposomes, and detergents are removed by dialysis or by treatment with detergent-absorbing resin. The purified receptor is capable of activating G protein upon binding of the agonist CP-55,940 [24]. The assay is very sensitive and as little as 2-3 ng of the receptor can be used per sample. The rates of activation are, obviously, affected by the orientation of the reconstituted receptor in proteoliposomes. In POPC/POPS bilayers the receptor is reconstituted in a random orientation so that only ∼50% of CB_2_ molecules are available for interaction with G protein [24]. Unlike proteoliposomes, in a typical *E. coli* membrane preparation the C-terminal part of CB_2_ appears to be fully accessible for interaction with the large MBP- TEV protease fusion providing indirect evidence for its accessibility for G protein binding [24].

The rates of G protein activation are affected by the composition of lipid matrix of proteoliposomes. The presence of ∼50% of phospholipids with negatively charged headgroup (i.e. POPS) in lipid bilayers results in the highest activation rate, presumably through more efficient stabilization of the receptor in active conformation [34]. The effect of the negative charge of phospholipids on activation rate of G protein appears to be receptor-specific since in the case of another class A GPCR, recombinant neurotensin receptor, ∼100% content of negatively charged POPG resulted in the highest rates of activation of G protein [54].

## Concluding remarks

We reviewed the methodology for expression, purification, stabilization and functional characterization of human cannabinoid receptor CB_2_. Expression of CB_2_ in *E. coli* as an N-terminal fusion with MBP yields fully functional receptor localized to the cytoplasmic bacterial membranes. Small affinity tags at the opposing ends of CB_2_ allow efficient purification with high recovery. Due to poor stability of the receptor in detergent micelles special care should be taken to preserve the functional fold of CB_2_. This is achieved by adding high affinity cannabinoid ligands and the cholesterol derivative CHS to detergent micelles during chromatographic purification of CB_2_. Furthermore, the addition of the cannabinoid ligand to the growth medium in the course of induction of CB_2_ synthesis significantly increases the yield of the functional receptor.

Long-term stability of CB_2_ is achieved by reconstituting the protein into lipid bilayers in a form of small spherical particles (proteoliposomes). The proteoliposomes are amenable to characterization by ligand binding and G protein activation assays. The structure and function of the receptor imbedded in lipid bilayers can then be studied by a variety of biophysical techniques including fluorescence-, CD, EPR, and NMR spectroscopy.

## Abbreviations

GPCR, G protein-coupled receptors; MBP, maltose-binding protein; Trx, thioredoxin; CHS, cholesteryl hemisuccinate; DDM, *n*-dodecyl *β*-D-maltoside; POPC, 1-palmitoyl-2-oleolyl-*sn*-glycero-3-phosphocholine; POPS, 1-palmitoyl-2-oleoyl-*sn*-glycero-3-phospho-L-serine; DPC, dodecyl phosphocholine; Ni-NTA, nickel-nitrilotriacetic acid; S-γ-GTP, guanosine-5’-3-O-(thio)triphosphate.
